# Tetanus

**Published:** 1877-07

**Authors:** N. F. Brown


### ARTICLE V.

### *Tetanus.*

BY N. F. BROWN, M. D.

A paper read before the Detroit Academy of Medicine.

This is a disease which has perhaps baffled the skill of the learned of the profession, in regard to its pathology and treatment, as much as any in the catalogue of diseases.

Tetanus is defined as a functional affection of the muscular system, in which there is continued rigidity of the muscles, accompanied with spasms, generally of a tonic character. Tetanus frequently arises from wounds or injuries, when it is designated as traumatic tetanus; occurring otherwise, it is termed idiopathic tetanus.

The important varieties of this disease are the traumatic and idiopathic. The latter variety is seldom seen.

Tetanus occurs in all ages, from early infancy to old age, but authors generally agree that it attacks the younger members of community, and that it is usually of the traumatic character.

Climate seems to exert a decided influence upon the disease, cases occurring much more frequently in warm and tropical regions than in temperate or cold latitudes. The causes of the disease are numerous, but prominent among these are wounds, particularly lacerated wounds; and it is worthy of notice that tetanus frequently results from a very slight wound, and that extensive lacerations are not so apt to be followed with the disease. All constitutions are susceptible to the disease, but generally the subject is one of debility, yet in hot climates a robust laborer is as apt to have tetanus from a slight wound as his weaker companion.

Th diagnosis is generally easily made, but the physiological effects of strychnia may be mistaken for tetanus.

When we look at a case of tetanus, we are astounded at the unknown morbific agent, or agents, which produce this terrible condition; and as post mortem examinations have thrown but little light on its pathology, we are compelled to treat the disease impirically. As I am not able to add anything to the literature of this subject, I will report upon a case of the traumatic variety which came under my care a few months since.

In the evening of the 28th of last September I was called to see a German, married, thirty-six years of age, light, wiry frame, nervous temperaments, and exceedingly irritable temper.

This man, although a "hard drinker," was industrious, and could work at anything from "roust-about" to blacksmithing and carriage making, and while thus employed, tramped upon a rusty nail, which passed through the sole of the shoe and entered the great toe, passing through close to the bone. After removing the nail he bathed the toe with cold water, and as the wound was not painful, continued work, and after one day the discharge ceased, the wound closed up; but on the fourth day it became painful and slightly inflamed, and on the fifth day he complained of aching in the lower extremities and "stiffness" in the neck and back.

In the evening, when I called, I found him sitting in a chair, perfectly rational, but quite sensitive and anxious; complained some of sore throat, but muscles of the face were not yet affected, but movements of the body caused great pain in the posterior muscles of the neck and back.

I gave chloral and bromide of potassium every hour in doses of twenty grains, but he was not able to take more than three doses, as trismus took place shortly after taking the third dose. In the morning I found him in true tetanic spasm, and all the muscles of the trunk and extremities seemed to be involved, and the body inclined to opisthotonos, the rigidity of the muscles being so severe that the body could have been wholly raised by lifting at either extremity. Deglutition being almost impossible, as even the contemplation of food or drink would induce severe spasm, I gave one grain of sulphate of morphia hypodermically, which had the effect of decidedly ameliorating the suffering of the patient.

He regained the use of his arms for a short time, which, previous to this, were immovable. During this time of relief considerable nourishment was taken in a liquid form, and the patient became quite cheerful, and expected to recover. He also took large doses of quinine, bromide of potassium, and belladona during this time, but after about six hours of partial relief, the spasms became more frequent and severe, and swallowing being impossible, I again gave morphia subcutaneously, but without decided effect.

The mind of the patient continued perfectly clear, but the suffering was indescribable, and continued till the fifth day, when there was considerable diminution of pain and spasm, lasting about one hour, when the patient gave a terrible shriek, the body assuming the position of emprosthotonos, which continued a few moments, and death came to the relief of the sufferer.—*Detroit Med. Journal.*

---

